# Hemodynamic changes of abdominal organs after CT colonography with transrectal administration of CO2: evaluation with early-phase contrast-enhanced dynamic CT

**DOI:** 10.1007/s11604-021-01125-5

**Published:** 2021-05-08

**Authors:** Kenichiro Ihara, Hideko Onoda, Masahiro Tanabe, Akihiko Kanki, Katsuyoshi Ito

**Affiliations:** 1 Department of Radiology, Yamaguchi University Graduate School of Medicine, 1-1-1 Minami-Kogushi, Ube, Yamaguchi 755-8505 Japan; 2 Department of Diagnostic Radiology, Kawasaki Medical School, 577 Matsushima, Kurashiki, Okayama 701-0192 Japan

**Keywords:** CT colonography, Hemodynamic, Hepatic pseudolesion

## Abstract

**Purpose:**

To evaluate the hemodynamic changes in the liver, pancreas, gastric mucosa and abdominal vessels in early-phase dynamic contrast-enhanced (DCE) CT immediately after CT colonography (CTC) with carbon dioxide expansion.

**Materials and methods:**

This study included 82 patients with DCE-CT after CTC (CTC group) and 77 patients without CTC (control group). Contrast enhancement values of the gastric mucosa, liver, pancreas, portal vein (PV), splenic vein (SpV), superior mesenteric vein (SMV), and inferior mesenteric vein (IMV) in early-phase CT were measured. The presence of hepatic pseudolesions were also recorded.

**Results:**

The mean contrast enhancement values of the gastric mucosa, pancreas and SpV in the CE-CTC group were significantly lower than those in the control group (*p* < 0.001, *p* < 0.001, *p* = 0.014). Conversely, the mean contrast enhancement values of the liver, PV, SMV and IMV in the CE-CTC group were significantly higher than those in the control group (*p* = 0.003, *p* = 0.013, *p* < 0.001, *p* < 0.001). Hypovascular hepatic pseudolesions were seen in early-phase CT in six patients after CTC, while they were not seen in the control group.

**Conclusions:**

On DCE-CT performed immediately after CTC with carbon dioxide expansion, it is important to be aware of the imaging findings induced by visceral hemodynamic changes.

## Introduction

Computed tomography (CT) colonography (CTC) has been widely used as a minimally invasive, reliable diagnostic technique for colorectal cancer [1–5]. CTC allows for the detection of colorectal cancers and polyps with high sensitivity, determination of local progression for staging, and depiction of synchronous lesions in the colon, even in patients with endoscopic inaccessibility. With standard CTC as a screening examination, the routine use of contrast medium is not necessary.

However, in patients with colorectal cancer, the preoperative detection of metastases is extremely important for deciding on the therapeutic strategy. Positive or negative extra-colonic findings can be as valuable as colonic findings in the management of patients with colorectal cancer [6, 7]. Therefore, the combination of CTC and dynamic contrast-enhanced (DCE) CT of the body performed immediately after CTC is considered efficient for the further investigation of both colonic and extra-colonic findings.

However, in DCE-CT performed immediately after CTC with carbon dioxide expansion, it is likely that hemodynamics of the abdominal organs and vessels will be affected by carbon dioxide absorbed through the colonic wall, possibly causing visceral enhancement alteration and perfusion-related hepatic pseudolesions. However, few previous reports have assessed the hemodynamic changes in the abdominal organs in DCE-CT performed after CTC.

Hence, the purpose of this study was to elucidate hemodynamic changes of the abdominal organs and vessels and the presence of perfusion-related hepatic pseudolesions in early-phase DCE-CT immediately after CTC with carbon dioxide expansion, comparing DCE-CT combined with and without CTC.

## Materials and methods

### Study population

Our Institutional Review Board approved this retrospective study and waived the requirement for informed consent. DCE-CT immediately after CTC with carbon dioxide expansion was performed in 106 patients from October 2010 to September 2019 (CE-CTC group). Among these patients, those with the following were excluded: retention of oral contrast agents in the gastrointestinal tract causing artifacts (*n* = 11); a history of gastrectomy (*n* = 3) or splenectomy (*n* = 1); presence of gastric diseases (*n* = 4) or huge liver metastasis (*n* = 1) affecting gastric mucosa or hepatic enhancement; heart failure (*n* = 1); the development of obvious collateral pathways such as gastric varices caused by portal vein hypertension (*n* = 1); and insufficient colon expansion (*n* = 2). Ultimately, 82 patients were included in the CE-CTC group. These included patients with colon cancer (*n* = 43), inflammatory bowel diseases (Crohn disease; *n* = 12, ulcerative colitis; *n* = 9), colon polyp (*n* = 2), colon adenoma (*n* = 2), diverticulitis (*n* = 2), and miscellaneous (*n* = 12).

DCE-CT immediately after CTC was performed to investigate extra-colonic abnormalities in patients with suspected malignant colonic diseases. In 16 of these 82 patients, DCE-CT without CTC had already been performed, namely five patients were performed within 1 month before CTC, while 11 patients were performed within 10 months after CTC. Thus, we additionally selected 66 age- and gender-matched patients randomly who underwent DCE-CT of the abdomen without CTC using the same contrast enhancement techniques during the same research period as the control group. Among these 66 patients, five were excluded because of the presence of obvious collateral pathways. The remaining 61 patients underwent DCE-CT for the further evaluation of suspected abdominal diseases such as hepatocellular carcinoma (*n* = 10), fever of unknown origin (*n* = 3), hematuria (*n* = 3), adrenal lesions (*n* = 3), malignant lymphoma (*n* = 2), liver metastasis (*n* = 2), benign hepatic lesions (*n* = 2), for the screening of malignancies (*n* = 8), and miscellaneous (*n* = 28). Ultimately, 77 patients in the control group were also included in this study. Chronic liver injury was found in four patients (chronic hepatitis B; *n* = 2, chronic hepatitis C; *n* = 1, alcoholic hepatic disease; *n* = 1) in the CTC group and 15 patients (chronic hepatitis B; *n* = 2, chronic hepatitis C; *n* = 10, fatty liver disease; *n* = 3) in the control group.

The CE-CTC group included 48 men and 34 women 21–85 years old (mean, 60 years), and the control group included 46 men and 31 women 20–84 years old (mean, 60 years).

### Imaging technique

CT was performed on a multidetector CT (MDCT) unit (Optima CT660 Pro, GE Healthcare or LightSpeed Ultra 16, GE Healthcare, Tokyo, Japan). The imaging parameters were as follows: tube voltage of 120 kVp, tube current of 180–250 mA, pitch 1.375 or 1.75, collimation 20 mm, and matrix, 512 × 512. The direction of all scans was craniocaudal. All patients received 600 mgI/kg body weight nonionic contrast material (iopamidol [Oypalomin 300 or 370, Konica Minolta, Tokyo, Japan and Iopamiron 370, Bayer, Osaka Japan]; or iohexol [Omnipaque 300, Daiichi Sankyo, Tokyo Japan]; or iomeprol [Iomeron 300 or 350, Eisai, Tokyo Japan]). The contrast material was administered at a rate of 3.3–5.0 mL/s using a mechanical power injector (Dual Shot; Nemotokyorindo, Tokyo Japan). Because a fixed injection duration of 30 s was used, the injection rate was automatically decided according to the patients’ weights. The contrast medium was injected through a 20- or 22-gauge plastic intravenous (IV) catheter placed in an antecubital vein. The section thickness and reconstruction interval were 5 mm. Total scan numbers of CTC and dynamic CT were four (two times scans for CTC with supine and prone position, and two times scans for dynamic contrast CT with early and late phases), and averaged estimated total radiation dose (CTDIvol) to the patients was 37 mGy. In the CE-CTC group, colonic distention in CTC was achieved using an automatic low-pressure with carbon dioxide delivery device (HP-2^®^; Horii Pharmaceutical Industry, Osaka, Japan) via a flexible rectal catheter. At first, unenhanced CT images were obtained for the purpose of CTC. DCE-CT during the early and late phases was then performed in all patients. Early- and late-phase images were obtained with delays of 40 and 210 s, respectively.

### Image analyses

The CT values of the gastric mucosa, liver (right lobe and left lobe), tail of pancreas, portal vein (PV), splenic vein (SpV), superior mesenteric vein (SMV), and inferior mesenteric vein (IMV) were measured in unenhanced and early-phase CT, respectively. The CT values of the pancreatic head were not measured, because they could be affected by the flow from both celiac artery and superior mesenteric artery. The contrast enhancement value was calculated as the difference in Hounsfield unit values between the unenhanced image and early-phase CT image. These measurements were conducted by two radiologists with 4 and 18 years of experience in abdominal CT interpretation blinded to the clinical data on a workstation (EV InsiteS; PSP Corporation, Tokyo, Japan) where patients’ information was anonymized, and CT data sets were randomized for blind-reading purpose.

The two radiologists set a circular or oval region of interest (ROI) on the images of each organ and vessel. The average contrast enhancement values of the two observers were calculated. The presence of hepatic pseudolesions was also recorded by collaboration. Hepatic pseudolesions were defined as hyper- or hypo-attenuating areas at the posterior edge of the left medial segment or surrounding the gallbladder fossa on early-phase CT. True lesions such as cyst, hemangioma and metastasis in these locations were carefully excluded based on unenhanced and late-phase CT findings.

### Statistical analyses

All data were analyzed using the JMP Pro software program (JMP Version 14; SAS Institute Inc., Cary, NC, USA). The Mann–Whitney *U* test was used to compare the mean contrast enhancement value in each organ and vessel, and the chi-square test was used to compare the presence of hepatic pseudolesions between the CE-CTC and control groups. A *p* value of < 0.05 was considered to indicate a statistically significant difference.

## Results

**Fig. 1:**
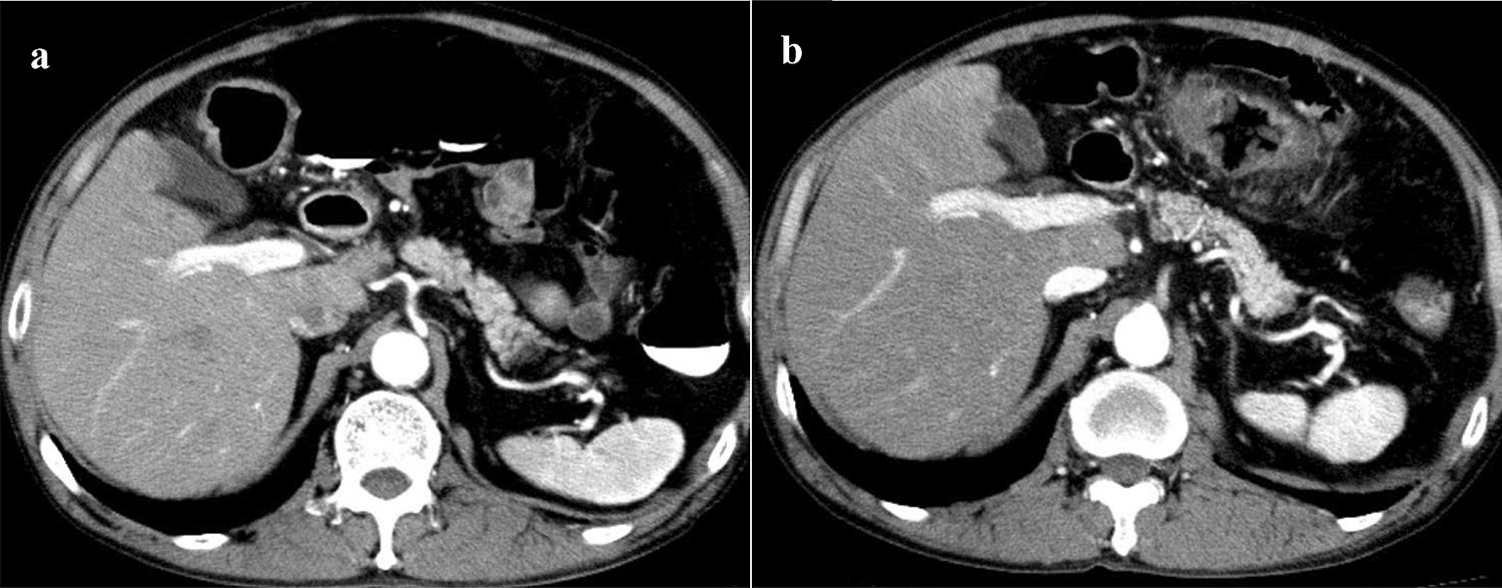
Early-phase CT (**a**) obtained after CTC with carbon dioxide expansion (CE-CTC), and early-phase CT (**b**) obtained without CTC procedures (control) from a same 73-year-old man. The contrast enhancement effect of the liver parenchyma in the early-phase CT obtained after CTC with carbon dioxide expansion was higher than that obtained without CTC procedures

**Fig. 2:**
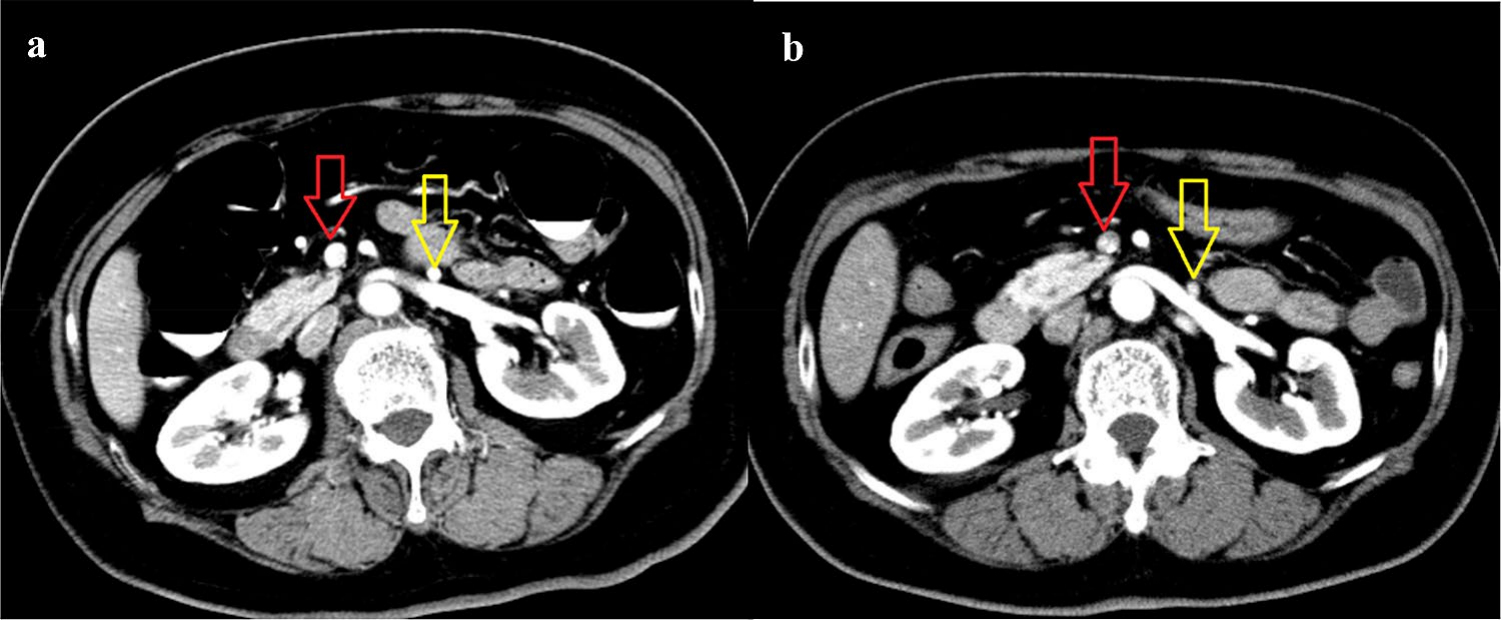
Early-phase CT (**a**) obtained after CTC with carbon dioxide expansion (CE-CTC), and early-phase CT (**b**) obtained without CTC procedures (control) from a same 63-year-old woman. The contrast enhancement effect of the SMV (red arrow) and IMV (yellow arrow) in the early-phase CT obtained after CTC with carbon dioxide expansion was higher than that obtained without CTC procedures. Dilatation of the SMV and IMV in a) was also observed

**Fig. 3:**
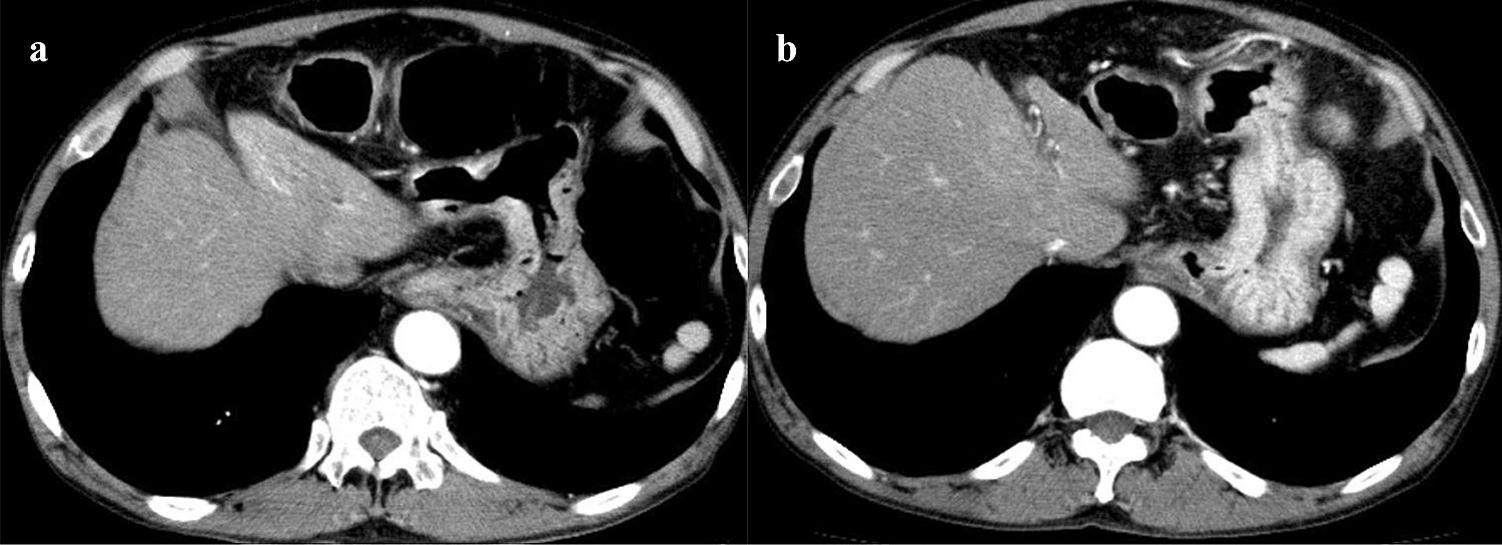
Early-phase CT (**a**) obtained after CTC with carbon dioxide expansion (CE-CTC), and early-phase CT (**b**) obtained without CTC procedures (control) from a same 73-year-old man. The contrast enhancement effect of the gastric mucosa in the early-phase CT obtained after CTC with carbon dioxide expansion was lower than that obtained without CTC procedures

The comparisons of the mean contrast enhancement value in each organ and vessel between the CE-CTC group and the control group are summarized in Table 1. The mean contrast enhancement values of the right and left hepatic lobes, PV, SMV and IMV in the CE-CTC group were significantly higher than those in the control group (33.9 ± 16.1 vs. 26.6 ± 16.0 HU, *p* = 0.003; 41.1 ± 18.0 vs. 33.1 ± 17.8 HU, *p* = 0.003; 155.1 ± 37.2 vs. 139.2 ± 46.7 HU, *p* = 0.013; 139.4 ± 53.1 vs. 98.9 ± 55.9, *p* < 0.001; 162.6 ± 55.0 vs. 65.7 ± 56.3, *p* < 0.001, respectively) (Figs. 1 and 2). Conversely, the mean contrast enhancement values of the gastric mucosa, tail of pancreas and SpV in the CE-CTC group were significantly lower than those in the control group (53.8 ± 19.4 vs. 73.5 ± 24.1 HU, *p* < 0.001; 69.81 ± 18.69 vs 83.08 ± 20.95 HU, *p* < 0.001; 163.3 ± 38.5 vs. 186.3 ± 48.3 HU, *p* = 0.014, respectively) (Fig. 3).

**Table 1:** Comparison of mean contrast enhancement CT values of each organs and vessels

Organ or vessel	Mean contrast enhancement values (HU)		
	CE-CTC group	Control group	*p* value
Right hepatic lobe	33.94 ± 16.06	26.55 ± 15.97	0.003
Left hepatic lobe	41.06 ± 18.03	33.05 ± 17.79	0.003
Gastric mucosa	53.78 ± 19.39	73.46 ± 24.12	< .0001
Tail of pancreas	69.81 ± 18.69	83.08 ± 20.95	< .0001
PV	155.1 ± 37.23	139.24 ± 46.69	0.013
SMV	139.42 ± 53.08	98.91 ± 55.94	< .0001
IMV	162.64 ± 54.95	65.72 ± 56.27	< .0001
SpV	163.31 ± 38.46	186.31 ± 48.26	0.014

**Fig. 4:**
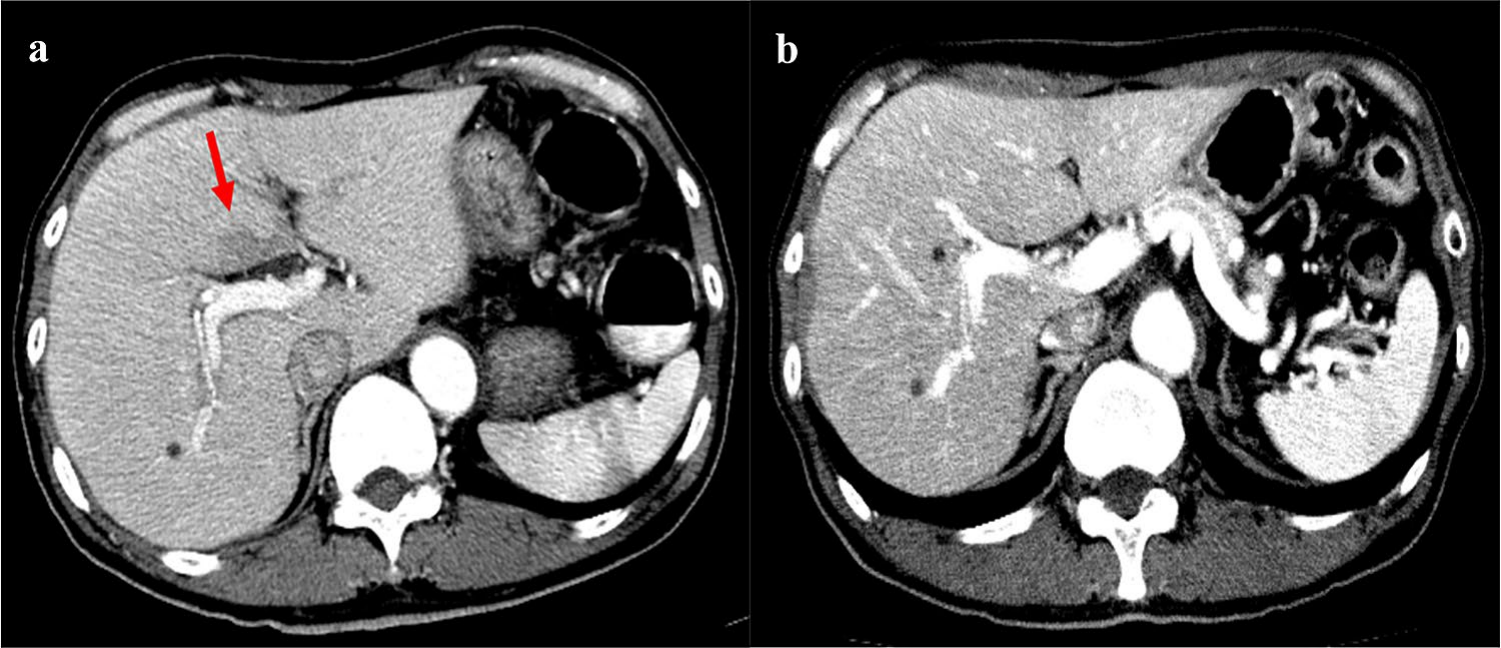
Hypovascular pseudolesion observed in a 58-year-old man. Early-phase CT (**a**) obtained after CTC with carbon dioxide expansion (CE-CTC) showed a hepatic pseudolesion seen as a hypo-attenuating area at the posterior edge of the left medial segment (arrow). On the early-phase CT (**b**) obtained without CTC procedures, a hepatic pseudolesion was not demonstrated. The contrast enhancement value of the liver parenchyma after CTC was higher than that without CTC procedures (right lobe: 66 vs. 51, left lobe: 75 vs. 67)

Hepatic pseudolesions were seen as hypo-attenuating areas on early-phase CT in six patients (7%) of the CE-CTC group at the posterior edge of the left medial segment (*n* = 5) or the surrounding gallbladder fossa (*n* = 1) (Fig. 4) while they were not seen at all in the control group (*p* = 0.016). Table 2 shows the results of the comparison of the mean contrast enhancement values of the liver between the CE-CTC patients with and without hepatic pseudolesions. The mean contrast enhancement values of the right and left hepatic lobes in the CE-CTC patients with hepatic pseudolesions were significantly higher than those in the CE-CTC patients without hepatic pseudolesions (51.8 ± 11.6 vs. 32.5 ± 15.1 HU, *p* = 0.005, 62.9 ± 13.8 vs. 39.3 ± 16.7 HU, *p* = 0.005, respectively).

**Table 2:** Comparison of mean contrast enhancement CT values of the liver between the CE-CTC group with and without hepatic pseudolesions

	Mean contrast enhancement values (HU)		
	Hepatic pseudolesion		
	Positive (*n* = 6)	Negative (*n* = 76)	*p* value
Right hepatic lobe	51.81 ± 11.6	32.5 ± 15.1	0.005
Left hepatic lobe	62.9 ± 13.8	39.3 ± 16.7	0.005

## Discussion

In the present study, the mean contrast enhancement CT values of SMV and IMV were significantly higher in the CE-CTC group than in the control group, suggesting an increased venous return from the large intestine. In addition, the mean contrast enhancement CT values of the liver were significantly higher in the CE-CTC group than in the control group, suggesting an increased portal venous flow, probably due to the early venous return from the SMV and IMV. Although the precise mechanism remains unclear, these findings may have been caused by carbon dioxide expansion of the colon during CTC.

Automated carbon dioxide insufflation is commonly used during CTC procedures, significantly improving colonic distention [8, 9]. Carbon dioxide is readily absorbed through the colonic wall approximately 20 times faster than room air because of a steep diffusion gradient [10]. Absorbed carbon dioxide affects the vascular smooth muscle of blood vessels in the colonic wall, causing arterial and venous vasodilation. Although several mechanisms of vasodilatation effect of carbon dioxide have been proposed, the major mechanism is considered to be related to a direct effect of extracellular H^+^ on vascular smooth muscle, causing a decrease in the pH and the subsequent reduction of free Ca^2+^ [11]. Therefore, it is speculated that vasodilatation was induced in the blood vessels of the large intestine that absorbed carbon dioxide and, as a result, the increased early venous return to the liver through the SMV and IMV was thereby promoted. In contrast, the mean contrast enhancement CT values of the gastric mucosa, tail of pancreas and splenic vein were significantly lower in the CE-CTC group than in the control group, suggesting that the blood flow in the celiac artery system such as gastric and splenic circulation in the CE-CTC group was complementarily reduced due to the increase in the mesenteric vascular circulation. While the routine use of contrast agents in CTC is not necessary for screening examinations, performing contrast-enhanced CT after CTC can be justified by the need for information about intra-abdominal organs other than colon. Therefore, it is essential to understand the changes in the contrast enhancement effect of the abdominal organs after CTC.

In the present study, hepatic pseudolesions were seen as hypo-attenuating areas at the posterior edge of the left medial segment or surrounding the gallbladder fossa on early-phase CT in six patients (7%) of the CE-CTC group, a significantly higher frequency than in the control group. The posterior edge of the left medial segment or surrounding the gallbladder fossa have been the characteristic locations of hepatic hypervascular pseudolesions sometimes observed on early-phase contrast-enhanced CT [12] or MRI [13, 14], angiography [15] or CT during arterial portography [16]. In early-phase CT and MRI immediately after IV contrast injection, these pseudolesions were seen as hyper-enhanced areas. These hypervascular pseudolesions are attributed to early drainage from a “third flow” source, such as cholecystic or aberrant gastric venous systems. It is likely that the blood flow from the cystic or aberrant gastric veins drains more rapidly into the sinusoids of the focal liver through the anatomical portal-systemic shunts than the flow from the vessels of the usual portal venous system, such as the superior mesenteric and splenic veins [13, 17].

However, the hepatic pseudolesions seen after CE-CTC in our study at the posterior edge of the left medial segment or surrounding the gallbladder fossa showed hypo-enhancement compared with the surrounding liver parenchyma. In CTC with carbon dioxide expansion, the liver parenchyma showed increased enhancement due to the increased early portal venous return from the SMV and IMV, and as a result, pseudolesions were more likely to appear as areas of relative hypo-enhancement, because they received a decreased blood flow from the third inflow. Similar findings may be seen on CT during arterial portography, where pseudolesions appears as areas of diminished portal perfusion (hypo-enhancement). One advantage of CTC over colonic endoscopy is that extra-colonic findings, including the preoperative detection of metastases, may strongly influence the accurate therapeutic management of patients with suspected colonic malignancies [7, 18, 19]. Therefore, being aware of the presence of these perfusion-related hypo-enhanced pseudolesions in characteristic locations on CE-CTC with carbon dioxide expansion will be clinically important for differentiation from true metastatic lesions.

Several limitations associated with the present study warrant mention. First, this was a retrospective study, and there might have been a potential risk of selection bias. Second, images from different CT machines were included. However, to minimize variability among different CT scanners, a uniform contrast injection protocol was used. Third, individual differences in hemodynamics and the duration of colon distension are unavoidable, even though we used the same imaging protocol. Fourth, in this study, there were no patients with hepatic pseudolesions in the control group, but it might be necessary to evaluate large number of patients to clarify the more exact frequency of hepatic pseudolesions. Finally, pathologic proof was not obtained in any patients with hepatic pseudolesions on CE-CTC with carbon dioxide expansion. However, the location of these pseudolesions was already well-known, so a percutaneous biopsy would not have been practical.

In conclusion, on DCE-CT performed immediately after CTC with carbon dioxide expansion, it is clinically important to be aware of the imaging findings induced by hemodynamic changes, such as increased contrast enhancement of the liver, SMV and IMV; decreased enhancement of the gastric mucosa; and the presence of perfusion-related hypo-enhanced hepatic pseudolesions at the posterior edge of the left medial segment or surrounding the gallbladder fossa, probably due to the increased early venous return from the large intestine caused by carbon dioxide expansion.
